# ¡Pásale!: Gaining Entrance to Conduct Research and Practice With Recent Hispanic Immigrants: Lessons Learned From the COPAL Study

**DOI:** 10.3389/fpubh.2022.879101

**Published:** 2022-05-06

**Authors:** Daniel Wood Soto, Jennifer B. Unger, Monica Pattarroyo, Alan Meca, Juan Andres Villamar, Maria Fernanda Garcia, Seth J. Schwartz

**Affiliations:** ^1^Keck School of Medicine, University of Southern California, Los Angeles, CA, United States; ^2^Department of Psychology, University of Texas at San Antonio, San Antonio, TX, United States; ^3^Northwestern Medicine, Chicago, IL, United States; ^4^Education and Human Development, University of Miami, Coral Gables, FL, United States; ^5^Department of Kinesiology and Health Education, University of Texas at Austin, Austin, TX, United States

**Keywords:** acculturation, immigration, recruitment, retention, methodology

## Abstract

Given the rapidly changing political rhetoric and policies concerning immigration, and the likely impact of this rhetoric on immigrants' adjustment, it is essential to understand the experiences of recently arrived immigrant individuals and families. This article describes methods to recruit and retain recently arrived Hispanic families in longitudinal research and clinical practice. Barriers to continued engagement with recent-immigrant families include residential mobility, wariness toward authority figures (including researchers and practitioners), and unpredictable work schedules. These barriers can lead to challenges related to recruitment/engagement, logistics, establishing trust, and retention. This article describes decisions made, experiences, and lessons learned in a longitudinal study of Hispanic families in two cities. We also provide implications for clinical practice.

## Introduction

The field of family therapy has increasingly attended to issues of cultural diversity and immigration (1). Working with recent immigrant families requires close attention to cultural processes that affect family relationships as well as individual family members' adjustment outcomes. Immigration can disrupt and alter family dynamics (2). Young children may adjust and learn the new language more quickly than their parents, creating an acculturation gap (3) that shifts the balance of power within the family hierarchy. The practice of family therapy is closely informed by family-based etiological research [e.g., (4)]; therefore, it is important to conduct studies of recent immigrants to inform family therapy and associated treatment approaches.

Engaging and retaining recent immigrant families, both in research and in clinical practice, presents logistical challenges. This article outlines the recruitment and retention methods that we used in our longitudinal study of recently immigrated Hispanic families in Miami and Los Angeles. These methods can be used by family therapists and other practitioners as well as by researchers.

## Hispanic Immigration and Acculturation

Hispanics—individuals with ancestry in Spanish-speaking countries in the Americas—have been the largest and fastest growing immigrant group in the United States (U.S.) for more than 20 years (5). Hispanics now represent 18% of the U.S. population, accounted for more than half of all U.S. population growth between 2000 and 2017, and are projected to account for one-third of the entire U.S. population by 2050 (6, 7). Approximately three-quarters of U.S. Hispanics are either immigrants or children of immigrants (8)—meaning that experiences related to international migration and acculturation continue to impact the lives of these individuals and their families on a regular basis.

The relevance of acculturation and related processes—such as culturally related stressors—among Hispanics has increased recently. Anti-immigrant sentiment, especially against Mexicans, has a long history in the US. In addition to the longstanding political rhetoric characterizing Hispanic immigrants as criminals and as a drain on the economy [e.g., (9)], recent events have escalated these anti-Hispanic, xenophobic attitudes in the U.S. The proposal to build a wall along the U.S.-Mexico border, the pledge to deport millions of undocumented immigrants (most of whom are Hispanic), and the end of the wet foot/dry foot policy that allowed Cubans to stay in the U.S. have created an increasingly hostile environment for Hispanic immigrants (10). Further, separating undocumented parents and children at the U.S.-Mexico border has damaged children's cognitive, emotional, social, and physical development (11, 12). It is essential to investigate the effects of this hostile environment on mental and physical health outcomes, and on risky and protective behaviors, among Hispanic immigrant families. Our study examined the effect of immigration on family dynamics and health risk behaviors among Hispanic immigrant families, with the goal of informing improved family-based clinical interventions.

In this article, we review many of the challenges faced, decisions made, and lessons learned in a longitudinal study of Hispanic families in Miami and Los Angeles. These experiences may be relevant for practitioners (i.e., ways to circumvent barriers and reach Hispanic immigrant families in need of services) as well as for researchers. The remainder of this article is divided into four main sections: challenges in studying acculturative processes in recently immigrated Hispanic adolescents; a description of our study; a discussion of difficulties and solutions in recruiting and retaining recent immigrant families; and recommendations for future studies. Difficulties and methodological approaches we suggest here include: 1. Relying on the local communities to validate our study; 2. Evolution of “comadres” as a recruitment and retention model; and 3. Flexible assessment schedules to accommodate working families and transportation challenges. We also discuss implications of our findings for family-based prevention and treatment efforts.

## Challenges in Studying Acculturative Processes in Recently Immigrated Hispanic Adolescents

Because immigration is a family phenomenon [e.g., (13)], and parents' stressors and experiences can affect adolescents' health behaviors and outcomes (14, 15), research on health behaviors among Hispanic immigrant adolescents should include the perspectives of both the adolescents and their parents. Clinicians addressing adolescent behavior problems should also include parents in the treatment process (4). Longitudinal studies are ideal because acculturation involves change over time (16). It is important to distinguish the experiences of recent immigrants from those of long-term immigrants and U.S.-born Hispanics because recent immigrants undergo more rapid acculturative change (17, 18). Further, recent immigrants, who stand out because of their foreign accents and mannerisms, may be most vulnerable to the effects of xenophobia and a hostile context of reception (19). It is essential for both researchers and clinicians to attend to these challenges faced by recent immigrant families.

Communities in the U.S. vary in the extent to which they welcome immigrants and provide culturally appropriate services (20). Multisite studies can elucidate this variation and inform therapists about service needs and barriers faced by specific immigrant groups in specific receiving contexts (21, 22). Family therapists, researchers, and service providers may encounter quite different challenges depending on a given family's background, receiving location and context in the U.S., and extent of U.S.-culture acquisition and heritage-culture retention [see, e.g., (23)].

## The Copal Study

COPAL (Construyendo Oportunidades Para los Adolescentes Latinos) was a six-wave, two-site longitudinal study of recently arrived Hispanic adolescents and their families in Miami-Dade and Los Angeles Counties. Data were collected between summer 2010 and spring 2013. Although research with underserved minority or immigrant populations is difficult to conduct (24), in COPAL we retained more than 80% of our sample at two sites, and through six assessment waves conducted over more than 3 years.

### Why Miami and Los Angeles?

There is considerable diversity within the U.S. Hispanic population. Hispanic groups in the U.S. come from distinct cultures of origin and have different histories of colonization, immigration, acculturation, and reception in the U.S. (8). To understand these differences, we recruited Hispanics from two of the largest cities with the largest raw number (Los Angeles) and proportional representation (Miami) of Hispanic immigrants^1^ (25). Los Angeles and Miami are home to extremely different Hispanic populations (mostly Mexican, Guatemalan, and Salvadoran in Los Angeles vs. mostly Cuban, Colombian, Nicaraguan, and Venezuelan in Miami). These nationalities differ in terms of legal documentation, education, and socioeconomic status (26)—which likely leads to differences in the ways these groups are received in and acculturate to the U.S., as well as differences in acculturative stress and discrimination (27). Whereas, Los Angeles has been home to a considerable Mexican-origin population since before the city's founding (28, 29), the large Cuban contingent in Miami originated during the Castro Revolution in the late 1950s and early 1960s (30). Further, whereas until recently Cubans were granted legal residence in the United States immediately upon setting foot on dry land, Mexicans do not enjoy such an advantage—and indeed many Mexican parents and adolescents remain undocumented, further complicating research recruitment and retention efforts (31, 32). The U.S. government resettled Cuban refugees in Miami beginning in the late 1950s, and these refugees built Miami into a major city (33). Perhaps as a result, Cubans and Cuban Americans hold the vast majority of elected offices in the City of Miami and in Miami-Dade County. In Los Angeles, although Mexicans and Mexican Americans have increased their political and economic power in recent years, there is still a great deal of discrimination against Mexican-origin individuals in the Los Angeles area (34). There may therefore be more mistrust and difficulty engaging and retaining Mexican-origin families (as compared to Cuban-origin families) in research and practice settings. Family-based practitioners working in these two states may face quite different challenges in terms of engaging and retaining low-income Hispanic immigrant families—including undocumented families—in prevention or treatment programs (35). These site differences underscore the importance of sampling from multiple locations in order to capture as much of the diversity within the US Hispanic population as possible.

Although other Hispanic nationalities are also represented in the COPAL sample, the largest percentages for Miami and Los Angeles were Cuban and Mexican, respectively (61 and 70%, respectively). We used multiple sites to increase the diversity within our sample and to determine whether associations between cultural factors and behavioral outcomes would replicate across Hispanic subgroups. Our findings, as well as our lessons learned during the engagement process, are relevant to practitioners and researchers working with Hispanic populations.

It is also essential to consider current events that may influence participation in research. During the recruitment phase for COPAL in Los Angeles (summer/fall 2010), Spanish-language media coverage at that time focused on stories of recent immigrants being deported and harassed by police and other authorities in the Southwest. Further, Arizona's anti-immigrant racial profiling law, SB 1070, was passed in 2010 and sparked intense debate and protest among Hispanics (primarily Mexican Americans) throughout the Southwestern United States (36). Especially in light of current political rhetoric and increasing xenophobic attitudes toward Hispanic immigration, it is likely that additional discriminatory events and legislation may occur in the future. This hostile political climate may have contributed to many undocumented immigrants' reluctance to participate in our study, as described below—and similar trends may be observed among practitioners (1). It is therefore essential to consider the socio-political context and its effect on Hispanic families when planning recruitment and retention strategies.

## Difficulties and Solutions in Recruiting and Retaining Recent Immigrant Families

In COPAL, to study the acculturation experience from the start, we focused on recently immigrated families (those who had lived in the United States for 5 years or less). Given the potentially high proportion of undocumented immigrants and the possibility that new immigrant families would return to their countries of origin, we faced challenges in recruitment, logistics, establishing trust, and retention. Our solutions dovetail with observations from other sources, suggesting that methods used to recruit and retain white samples are generally not appropriate for recruiting recent immigrant families (37). As a result, alternative strategies are needed (24).

## Challenges in Recruitment and Retention

### Logistical Challenges in Project Development and Recruitment

Undocumented immigrants, in particular, can be difficult to track longitudinally—they change residences and phone numbers often to avoid immigration authorities, and they are often mistrustful of U.S. institutions (38). Further, the availability of a land border between California and Mexico allows Mexican immigrant families to travel back and forth between countries. At the time of this study, it was common for Mexican families in California to travel back and forth across the border to visit relatives or obtain healthcare (29, 39). A common pattern among the Mexican families in COPAL was for the adolescents to spend summer vacations with grandparents or other relatives in Mexico while their parents worked. These patterns also differentiate Mexican families from their Cuban counterparts, who rarely (if ever) return to live in Cuba following immigration, and who are unlikely to send their children to Cuba. This, and other, variations between the two sites led to differences in retention rates and necessitated somewhat unique research strategies. Practitioners working with recently immigrated Hispanic adolescents and families are also faced with challenges related to residential mobility and—in the case of undocumented immigrants—a desire to avoid detection. Increased anxiety, stress and depression associated with acculturation requires adaptations in engaging these communities (40). In addition to adding complexity to research efforts, these challenges may also increase the difficulty in providing services to recent Hispanic immigrants—and we hope that some of the insights we gained from COPAL will help practitioners and service providers to reach and serve this population.

### Identifying Recent Immigrants

In Miami, we were able to recruit recent-immigrant adolescents from (English for Speakers of Other Languages) ESOL classes. However, in Los Angeles, students are commonly transferred out of ESOL after 1 year, such that many recent immigrant adolescents would be in mainstreamed classes. This variation in ESOL policies increased ease of recruitment in Miami because most recent immigrant adolescents would be in ESOL classes. In Los Angeles, the policy limiting ESOL enrollment to 1 year required us to work closely with school administrators to identify recently immigrated students in mainstreamed classes. Working closely with school districts allowed us to assemble a list of contact information and directly contact recent immigrant Hispanic families. We followed the Federal Educational Rights and Privacy Act (FERPA) guidelines by partnering with our school districts on our research projects. According to FERPA, schools are allowed to share contact information with organizations conducting research with or on behalf of the school (41). Recruitment methods must be flexible enough to accommodate differing policies and procedures across sites. These policies can exist at the school, district, city, county, or state level, and it is important to be aware of any policies that can affect recruitment strategies.

### Trust in Universities Conducting Research

Within Miami-Dade County, the University of Miami is widely recognized as a trustworthy research institution and provides instant credibility even among recent immigrants. As we later learned, many of our target families in Los Angeles did not know about the University of Southern California and were mistrustful. Some families later told our staff that initially they thought our assessors were government agents and would attempt to deport them. Researchers and practitioners should not necessarily assume that their university or healthcare institution will be well-known within the recent immigrant community.

## Challenges in Establishing Trust and Gaining Entry

Initially, we anticipated that it would be easier to recruit 150 recent-immigrant families in Los Angeles than in Miami, simply because of the larger number of Hispanics in Los Angeles County. However, this assumption was incorrect. Regardless of the fact that the Los Angeles team employed primarily Mexican assessors and the Miami team employed primarily Cuban, Colombian, and Nicaraguan assessors, greater proportions of families in Los Angeles than in Miami declined to participate. The Miami team approached 500 families to recruit 150, whereas the Los Angeles team approached 900 families to recruit 150. Los Angeles families were highly skeptical of our study team and of our motivations for conducting a study on recent immigrants. One mother considered cutting off telephone service and moving after our recruitment team contacted her from our phone list. She was only willing to participate after her neighbor informed her that she had also taken the COPAL survey and that she would not suffer any adverse consequences from participating. Such events, although less common, did occur in Miami. One father circled the research center building several times before parking. He appeared quite anxious, and when an assessor attempted to conduct the consent discussion, the father abruptly ended the discussion and quickly left the building. Therefore, experiences of skepticism and mistrust should be expected given the often anti-immigrant sentiments among large segments of the US population. When these types of experiences occur, it may be beneficial to enlist other families already enrolled in the study to attest to the legitimacy of project and the team.

In our consent discussions with families, we emphasized information about confidentiality and the benefits our study would bring to the community. Tapping into the intrinsic value of participation alleviated much of the concern and mistrust among our study families. All of our assessors were from the same communities where participants lived. In some cases, our assessors were from the same states and cities in Mexico as our study participants. The form of Spanish that our assessors used was formal (i.e., “usted” instead of “tú”) but was also friendly. It was not uncommon for Los Angeles assessors to begin using the “tú” form of Spanish once requested by participants. (This was less likely to occur in Miami, where families continued to use “usted”.). Assessors were often able to make connections with participants over the telephone by striking up “small talk” with parents—such as data collectors talking about their own hometowns, their children, and their own journeys to the United States. This small talk helped us to establish rapport with participants prior to meeting with families and initiating the consent process. Researchers and practitioners should therefore consider the importance of casual conversation in promoting trust among recent immigrant families.

## Retention Challenges

Immigrant families tend to move residences often, change phone numbers frequently, have unpredictable work schedules, and lack reliable transportation (42). Studies conducted using office-based assessments, and where assessment dates and times are arranged around researchers' convenience, are not well suited for the kinds of families who participated in the COPAL study. Similar principles may apply to clinical practice with recent immigrant families.

The first decision that we faced in COPAL was how often to conduct assessments. Although more frequent contacts can reduce attrition, they also can be overly burdensome to participants (24). Following Lerner et al. (43), we decided to assess twice per year. Despite the relatively short intervals between assessment timepoints, many of the COPAL families moved (and some moved multiple times) between timepoints. Residential mobility was more common in Los Angeles (10% moved at any point in the study) than in Miami (5%). To facilitate retention, we called each family at least once between assessment points. All of the calls were made by the *comadre* (Spanish speaking case workers who established relationships with study families) who had engaged the family and with whom they had an existing rapport.

We initially employed a team of Spanish speaking assessors for recruitment and data collection. At each site, families began to ask for, or otherwise prefer, communicating with a specific assessor. As a result, the assessment team model shifted where one assessor emerged as a face of the study for all participating families. Internally we labeled this lead assessor as the study “Comadre”. It is essential to designate such a comadre for each study family to communicate with in order to increase rapport and retention within the sample. *Comadres* are helpful in linking immigrants with primary care services (44) and mental health treatment (45), as well as research (46). In COPAL, *comadres* reached out to each family every 2 or 3 months to update contact information. At both sites, the same *comadre* was used throughout the study—resulting in an 85% retention rate through six timepoints over 3 years (including no attrition in Miami after Time 3, and only five Los Angeles families lost to follow-up after Time 3). Each *comadre* knew each family by name, and many families would not speak to anyone on the study team other than the *comadre* with whom they had developed a relationship. We also used the same assessors at both sites throughout the study.

*Comadres* and assessors took care to form relationships with study families during the recruitment phase by building rapport and spending time with families in culturally appropriate ways such as having dinner, helping to translate school documents, suggesting resources (e.g., doctors, employment services) for family members, and sharing a cup of café con leche. When the Obama administration issued a deferred-action executive order allowing undocumented immigrant adolescents and young adults to obtain driver's licenses and work permits as long as they had entered the U.S. before age 16, several Los Angeles families approached our study team and asked us to serve as a reference. Our Miami team referred one family to a local physician for a serious health problem and helped another family to coax their son back into school after he had stopped attending. Researchers and practitioners must consider these engagement and retention techniques in order facilitate relatively high and stable retention rates among a highly mobile, recent immigrant Hispanic population.

Many Los Angeles families had prepaid cell phone numbers that were changed or disconnected after the prepaid amount had been exhausted. Our strategy for handling these challenges was to ask each participating parent for the names and phone numbers of three “contact persons” who would be able to locate the participating parent in the event that we could not. We included text in our consent form requesting permission to reach out to these contact persons, and to ask for an updated list of contact persons at each assessment timepoint. We also asked for consent and assent to obtain three contact persons for adolescents. We therefore had six contact persons for each participating family. Because contact persons were often participants' friends or family members, they tended to be highly mobile as well, so it was essential to have several contact persons for each participating family (24).

### Need for Flexibility

Another lesson was that assessors needed to be available whenever and wherever necessary, and that assessors needed to exercise a great deal of patience. On many occasions, families would schedule assessment appointments but would not be present at the agreed-upon time and location. Many COPAL families worked multiple jobs and being called into work took precedence over keeping an assessment appointment. Assessors would often be disappointed or frustrated, and the project director or assessment coordinator would have to help them to recover their motivation. Holding regular meetings with the assessment team gave assessors the opportunity to express frustration, share successes, and talk about ways to desensitize oneself from the perceived rejection when a family did not come to a scheduled appointment (or was not home for a scheduled home assessment). Further, we learned that we needed to budget for up to twice as much staff time as we had originally anticipated given the number of canceled or missed assessment appointments.

The concept of punctuality (showing up and being on time for a scheduled appointment) varies greatly between primarily individualistic and primarily collectivistic cultural contexts (47). In highly individualistic contexts such as the US, being on time for scheduled appointments is highly valued, and being late (or not showing up at all) is considered rude. Conversely, in more collectivist contexts—especially those characterized by a strongly fatalistic cultural orientation (i.e., what will happen is out of our control and we simply have to accept it)—punctuality is a more flexible concept, and it is often acceptable to arrive hours late for an appointment or to miss it altogether. The U.S. and many Latin American countries are diametrically opposed on both individualism-collectivism and fatalism (48), suggesting that COPAL families likely placed far less importance on being on time than the research team did. We often found it useful to emphasize these cultural differences when helping research team members to recover their motivation following a series of missed assessment appointments.

Consistent with the theme of flexible punctuality, just as families could become *unavailable* without notice, they also sometimes became *available* without notice. Each study family had the *comadre's* cell phone number, and the *comadres* frequently received last-minute evening or weekend phone calls from families indicating that the family was home and ready to be assessed. The *comadre* would then have to travel to the research center to retrieve the assessment equipment (a laptop computer for the adolescent and a tablet PC for the parent), and then go to the family's home (or meet them at another convenient location, e.g., local library, community center, or school) to conduct the assessment. This often involved assessors having to change their evening or weekend plans to accommodate a family's sudden availability.

It is essential to accommodate families' preferences in terms of survey locations, and to be aware that these preferences often change over time. Miami families preferred to complete their assessments at the university research center or at the adolescent's school, but over the course of the study many Miami families began to prefer home visits. Most Los Angeles families were unwilling to come to the university research center and preferred to complete their assessments at local locations such as libraries and neighborhood community centers.

### Incentives

Providing incentives for participation is essential for maintaining interest and motivation. In COPAL, because the adolescents were minors, cash payments were given to parents. Parents received $40 for completing the baseline assessment, and this payment increased by $5 at each successive timepoint (up to $75 at the sixth timepoint). Each adolescent was given a movie ticket voucher in exchange for participating—though it should be noted that many adolescents did not use the tickets (possibly because they lacked access to a nearby movie theater or someone to accompany them to the movie), and a different incentive (such as a gift card) might have been more useful. The incentives were quite effective in recruiting low-income families, but they might be less effective with more affluent families.

## Recommendations for Future Studies

We offer specific recommendations for researchers and practitioners seeking to work with recent-immigrant Hispanics. First, in school-based samples, involving school personnel in the recruitment process can promote trust and engagement, because parents have an existing relationship with the school. The school personnel can help alleviate parents' fears that the research team is connected to immigration authorities or law enforcement. Because we were recruiting from ESOL classes, we worked directly with each school's ESOL coordinator/teacher In Los Angeles, we also engaged principals and assistant principals who helped us to identify recently immigrated adolescents who were not in ESOL classes. It is also helpful to offer incentives or thank-you gifts to school personnel who assist with recruitment.

Second, it is critical to hire staff who are from the same communities as the study respondents. *Being from the same ethnic group is not enough*. These individuals must speak in the same ways, be from similar SES brackets, and engage in similar cultural activities as the study participants. For example, when recruiting undocumented Mexican and Central American immigrant families, employing data collectors from the same parts of Mexico or Central America that the families are from, and who are familiar with the issues facing undocumented families, is important where possible. The ending of the wet foot/dry foot policy suggests that, moving forward, similar practices may be necessary with Cubans as well. For another example, in a Cuban or Dominican immigrant community where many individuals practice *santer*í*a* and other indigenous religions, data collectors might be other individuals from these same neighborhoods who also practice these same religions. Families must be comfortable working with data collectors and must share important commonalities with them. Similar principles apply to practitioners—immigrant families may be most willing to engage with practitioners (or with *comadres* working on behalf of practitioners) with whom they share cultural similarities.

Third, once staff members establish relationships with families, it is important to maintain staff continuity to foster those relationships. Although staff turnover is inevitable, it may be advisable to have the staff member who has maintained a relationship with the family call or visit them to introduce the person who will be replacing her/him on the research team. This may be especially true among new and/or undocumented immigrant families who understandably distrust phone calls from strangers. Our *comadre* approach was extremely effective in encouraging retention; families often *requested* that specific data collectors conduct the assessments with them. A similar approach may be advisable for practitioners who are conducting outreach into immigrant communities—maintaining personal relationships and trust with the person representing the provider is essential for keeping people engaged in service delivery activities.

Fourth, staff should be prepared to invest significant time, effort, and flexibility to maintain contact with immigrant families. Researchers should plan to conduct home visits or to meet participants at local public locations. Because of their unpredictable lives, immigrant families often miss scheduled appointments or reschedule at the last minute. Researchers should budget for sufficient incentives, mileage, and data collector time (taking into account the missed appointments, cancellations, and rescheduling that are almost certain to occur). Practitioners must leave room in their calendars for no-shows. Researchers—and practitioners—must take into account the differing value systems that recent immigrants may hold regarding punctuality and the importance of prioritizing previously scheduled appointments vis-à-vis other opportunities or obligations that might arise.

Fifth, when opportunities arise for the research team to serve as a resource for study families, the research team should capitalize on these opportunities. When DACA was enacted during our study, several adolescents requested letters of support to indicate that they had been living in the U.S. and had not been involved in criminal activities. Such positive contributions to the families' overall welfare, in the long run, were more valuable to families than movie gift cards or small amounts of cash. In essence, our research team did more than assess these families—they became active contributors to families' lives. Such active contributions may be especially important in times of increased xenophobia and hostility toward immigrants. It is important, however, to ensure that help provided by the research team does not compromise the data being collected.

Finally, the survey instruments need to be in as many formats as possible. Common formats include Internet, landline, paper, and smartphone/tablet. Many adolescents have access to smartphones and tablets and can complete surveys online using these devices. Parents, however, may be less familiar with these technologies. In COPAL, because some parents did not read well, we used an audio computer assisted interviewing [ACASI; (49)] system where parents could listen to the survey items and response choices, and then indicate their responses using a touch screen. Regardless of the assessment modality used, however, families may be most likely to complete the survey if rapport has been established with the research team.

## Conclusion and Implications for Clinical Practice

Conducting research and interventions with immigrant populations is important in helping us fully understand the immigrant experience and its effects on behavioral health. Our COPAL project focused on recently arrived Hispanic immigrants and presented us with a unique opportunity to adapt traditional recruitment and data collection methods for this difficult to reach population.

The difficulties and solutions for recruitment and retention varied by site, such as more mistrust of the study team among Los Angeles families than among Miami families. Our expectation that the Los Angeles site would have an easier time with recruitment because of a larger Hispanic population was completely contrary to our experience. These difficulties in recruiting may increase under conditions of widespread anti-immigrant hostility and xenophobia. Nonetheless, we were able to adapt our strategies and retain nearly 85% of the sample through six waves over 3 years. Almost all of the attrition from the study occurred during the first year (12 families in Miami and 23 families in Los Angeles). Between Times 3 and 6, only four families left the study.

One limitation of the current findings is that it may be somewhat cost prohibitive for smaller academic institutions and community organizations. As such, we suggest considering adaptations of the methods that we used here such as recruiting multiple cohorts of participants or spreading recruitment over a longer period of time so as not to overburden the smaller assessment team. Further, it is important to consider the extra effort and budget needed when planning a study with recent Hispanic immigrants. Finally, when hiring staff, one should consider flexibility of scheduling and the importance of establishing rapport with study participants. This will require extra staff effort which could increase study expenditures. It may be necessary to recruit volunteers and students who may be motivated by a desire to give back to their communities.

In family therapy with Hispanic immigrant families, practitioners must be informed by both theories and research findings on unique cultural factors of these families (50). Historically Hispanic families underutilize mental health services, due to lack of knowledge about services, language barriers, and perceived stigma (51). Having the support from *comadres* can encourage Hispanic immigrant families to seek out family therapy and other mental health services. Practitioners working with Hispanic immigrants must develop culturally informed practice that is sensitive to the diverse value systems and life experiences of diverse Hispanic immigrant families. Family practitioners can serve as a source of information and support for Hispanic immigrant families as they adapt to life in a new country. Researchers, practitioners, and community organizations working with recently arrived immigrant populations can capitalize on the lessons learned from our project to engage and retain Hispanic recent immigrant families—a highly underserved and understudied population.

## Data Availability Statement

The original contributions presented in the study are included in the article/supplementary material, further inquiries can be directed to the corresponding author/s.

## Ethics Statement

The studies involving human participants were reviewed and approved by University of Southern California Institutional Review Board. Written informed consent to participate in this study was provided by the participants' legal guardian/next of kin.

## Author Contributions

All authors listed have made a substantial, direct, and intellectual contribution to the work and approved it for publication.

## Funding

Preparation of this article was supported by Grant DA025694 from the National Institute of Drug Abuse and National Institute on Alcohol Abuse and Alcoholism.

## Conflict of Interest

The authors declare that the research was conducted in the absence of any commercial or financial relationships that could be construed as a potential conflict of interest.

## Publisher's Note

All claims expressed in this article are solely those of the authors and do not necessarily represent those of their affiliated organizations, or those of the publisher, the editors and the reviewers. Any product that may be evaluated in this article, or claim that may be made by its manufacturer, is not guaranteed or endorsed by the publisher.

## Footnotes

^1^Note that “Hispanic immigrants” does not include Puerto Ricans, who are U.S. citizens at birth.
